# Prevalence of Extended-Spectrum *β*-Lactamase-Producing *Enterobacteriaceae* in Ethiopia: A Systematic Review and Meta-Analysis

**DOI:** 10.1155/2021/6669778

**Published:** 2021-03-31

**Authors:** Teklehaimanot Kiros, Lema Workineh, Tegenaw Tiruneh, Tahir Eyayu, Shewaneh Damtie, Debaka Belete

**Affiliations:** Department of Medical Laboratory Sciences, College of Health Sciences and School of Medicine, Debre Tabor University, Debre Tabor, Ethiopia

## Abstract

**Background:**

Antimicrobial resistance especially caused by extended-spectrum *β*-lactamase-producing *Enterobacteriaceae* (ESBL-PE) has become a global public health concern. Globally, these isolates have remained the most important causes of several infections and associated mortality. Their rapid spread in Ethiopia is associated with a lack of regular surveillance and antibiotic stewardship programs. Isolates of ESBL-PE from different regions of Ethiopia were searched exhaustively. However, published data regarding the pooled estimate of ESBL-PE are not conducted in Ethiopia. For this reason, we systematically reviewed laboratory-based studies to summarize the overall pooled prevalence of the isolates recovered from various human specimens.

**Methods:**

An exhaustive literature search was carried out using the major electronic databases including PubMed, Web of Science, MEDLINE, EMBASE, CINAHL, Google Scholar, Cochrane Library, Scopus, and Wiley Online Library to identify potentially relevant studies without date restriction. Original articles which address the research question were identified, screened, and included using the PRISMA follow diagram. Data extraction form was prepared in Microsoft Excel, and data quality was assessed by using 9-point Joanna Briggs Institute critical appraisal tools. Then, data were exported to STATA 16.0 software for analyses of pooled estimation of outcome measures. Estimation of outcome measures at 95% confidence interval was performed using Der-Simonian-Laird's random-effects model. Finally, results were presented via text, figures, and tables.

**Results:**

A comprehensive electronic database literature search has yielded a total of 86 articles. Among the total, 68 original articles were excluded after the review process. A total of 18 studies with 1191 bacterial isolates recovered from 7919 various clinical samples sizes were included for systematic review and meta-analysis. In this study, the pooled prevalence of ESBL-PE was 18% (95% CI: 9–26). Nine out of the total (50%) reviewed articles were studied using the combination disk test. Likewise, *E. coli* and *K*. *pneumoniae* (50% both) were the predominant isolates of ESBL-PE in addition to other isolates such as *Salmonella* spp. and *Shigella* spp.

**Conclusion:**

This meta-analysis has shown a low pooled estimate of ESBL-PE in Ethiopia.

## 1. Introduction

Antimicrobial resistance (AMR) that could be caused by bacteria, virus, fungus, and parasite has remained the global public health problem [1]. The evolution of AMR significantly threatens the effective prevention and control strategies to a range of infections such as urinary tract infections, bloodstream infections, wound infections, and pneumonia due to the prevalence of drug-resistant bacterial strains which are responsible for healthcare-associated and community-acquired infections globally. Extended-spectrum beta-lactamase-producing *Enterobacteriaceae* (ESBL-PE), particularly the Gram-negative bacilli such as *Escherichia coli* and *Klebsiella pneumoniae*, are the major global health threat due to their pattern of multidrug resistance [2].

Poor drug regulation and control systems in many parts of the world have led to an extensive misuse and overuse of antibacterial drugs in both humans and animals settings [3]. Such activities collectively favor the spread of resistant bacterial strains into the community and the clinical settings that subsequently decrease the treatment outcome. This is because drug-resistant pathogenic bacterial strains are capable of proliferating and spreading anywhere when infection prevention and control measures are inadequate [2, 4]. ESBL-producing pathogens mainly the Gram-negative bacteria are posing great challenges to the global health community who engaged in the diagnosis, treatment, infection prevention, and control as well as the development of a new antimicrobial agent to tackle the grave impact of AMR [5]. Interventions aimed to mitigate the predisposing factors for acquisition of ESBL, complex treatment due to multiresistance, and none of the advanced diagnostic laboratory collectively hinders the quality of healthcare to combat ESBLs. As a result, these organisms have remained one of the most important agents of nosocomial, community-acquired infections, and foci of many clinical guidelines and research studies [6].

*Enterobacteriaceae*, which are Gram-negative, nonspore-forming facultative anaerobic bacilli, are important causes of nosocomial and community-acquired infections. Although ESBLs are predominantly found in *E. coli* and *Klebsiella* spp., other pathogens such as *Enterobacter* spp., *Proteus* spp., *Citrobacter* spp., *Morganella* spp., *Providencia* spp., and *Salmonella* spp. are also capable of producing ESBLs to cause dozens of infections. *β*-Lactamase enzyme production has remained the most common resistance mechanism against *β*-lactam antibiotics [7, 8]. This is because bacteria's *β*-lactamases are capable of inactivating the *β*-lactam antibiotics by hydrolysis that subsequently resulted in ineffective regimens as therapeutic agents. Later on, the pathogen will become resistant to various categories of *β*-lactam antibiotics such as cephalosporins, monobactams, and carbapenems. Unless and otherwise early screening prompt, ESBL-producing organisms will have serious tremendous consequences including therapy failure, laboratories detection, and infection control issues [9].

Beta-lactam antibiotics including extended-spectrum penicillins, cephalosporins, monobactams, and carbapenems are the predominant antibiotics used to treat infections caused by ESBL-PE. Despite beta-lactam antibiotics have known to be the most prescribed antibiotics by many clinicians, ESBL-PE is still causing several hospital and community-acquired infection worldwide. Nowadays, ESBL-PE is responsible for numerous outbreaks of infection posing challenging infection control issues, diminishing many clinical outcomes [7, 10]. Likewise, plasmid-mediated ESBL resistance among members of *Enterobacteriaceae* is also easily transmittable. In this condition, the choice of antimicrobial agents to treat infections will be limited [11].

Nowadays, many patients demand antibiotics such as carbapenems that subsequently led to the rapid selection of carbapenem-resistant pathogens. Hence, this increasing alarming rates of AMR mainly caused by Gram-negative bacteria are concerning for many reasons including increased hospital costs, therapeutic failure, prolonged hospital stay, and increment in mortality rates [12]. However, the lack of comprehensive and compiled nationwide study to estimate the magnitude of ESBL among the Ethiopian population is lacking. This is because the survey on ESBL-PE in the local scenario is fundamental to grasp the gap in the local clinical and epidemiology data. Besides, the local epidemiological data enable health experts to understand the burdens to implement breakthrough infection prevention and control strategies to mitigate community-acquired and nosocomial infection due to ESBL-producing pathogens. Therefore, this study aimed to systematically review the different studies conducted on ESBL-producing organisms and estimate the prevalence of ESBL-PE in Ethiopia using meta-analytical methods.

## 2. Methods

### 2.1. Study Setting and Design

This systematic review and meta-analysis study was conducted in Ethiopia, a country with the second most populous next to Nigeria in Africa. The current total population of Ethiopia is estimated greater than 115 million (https://worldpopulationreview.com/countries/ethiopia-population). Any laboratory-based studies that address the primary outcome of interest in light of the concept of the prevalence of extended-spectrum *β*-lactamase-producing *Enterobacteriaceae* investigated using the standard bacteriological approaches from the Ethiopian population were systematically studied. Consequently, a systematic review and meta-analysis study was conducted to sum-up the prevalence of bacterial isolates recovered from various human specimens published at any time frame without date restriction.

### 2.2. Literature Search Strategy

An exhaustive literature search strategy toward studies that reported prevalence of extended-spectrum *β*-lactamase-producing *Enterobacteriaceae* was conducted for grey and peer review literature with no date restrictions. Electronic databases search engines such MEDLINE, PubMed, Cochrane Library, Scopus, Google Scholar, EMBASE, CINAHL, Wiley Online Library, Index Medicus, and Web of Science were exhaustively searched to identify potentially published relevant studies. Expert consultation, reference tracing of potential full-text articles, preprints, and conference proceedings were carefully assessed to complete the search strategy. Moreover, additional data were sought even from the authors to complete the information through e-mail contact, especially for inaccessible/full of charge original research articles. Furthermore, regular alerts were established to few selected databases such as PubMed and Google Scholar to update the search strategy before the publication of this article. Moreover, Google and other Internet search engines were used to search for additional web-based or electronic materials. Hence, the searches were rerun just before the final data analyses.

The keywords used for the search and how relevant materials used for the review were selected by the authors. As a result, keywords were developed following the medical subject heading (MeSH) search strategy. Besides, the Boolean operators (AND, OR, and NOT) and wild cards (“∗”) were customized by the group of authors based on the outcome measures. The search strategy was made using keywords such as “Extended-spectrum beta-lactamase-producing isolates,” “Multidrug-resistant bacteria” OR “Antimicrobial resistance” AND “Ethiopia,” “ESBL producing *Enterobacteriaceae*,” “ESBL infections,” Extended-spectrum beta-lactamase-producing *Enterobacteriaceae*,” and “Prevalence” OR “epidemiology of ESBL” AND “Ethiopia.”

### 2.3. Eligibility Criteria

Before identifying appropriately published relevant full-text articles either in local or international journals, a selection criteria checklist for study eligibility was developed by the authors.

*Inclusion Criteria*: All studies which met at least the following criteria were included in the review process. These were [1] studies that reported the prevalence of ESBL-PE in any region of Ethiopia [2], a study that was conducted on human/clinical specimens [3]. It accurately reports the bacterial isolates of *Enterobacteriaceae* including their drug susceptibility/resistance tested against to at least amoxicillin plus clavulanic acid and other third-generation cephalosporins based on the CLSI [13] guideline [4], and studies with confirmed ESBL using phenotypic detection methods and that used molecular techniques for ESBL gene variants detection [5]. It reported the numbers of ESBL-producing isolates of *Enterobacteriaceae*, and [6] all relevant free of charge full-text original research articles that were published in English either locally or under international journals were included in the review process.

*Exclusion Criteria*: The study was excluded if [1] there is no confirmation of ESBL production using phenotypic and/or genotypic methods such as the double-disc synergy test (DDST), the combination disk test (CDT), minimum inhibitory concentration (MIC), the epsilometric test (E-test), and molecular methods such as polymerase chain reaction (PCR) [2], if with incomplete information regarding the primary goal or outcome measure of the study [3], and studies performed outside Ethiopia [4]. Research articles which were completely irretrievable through the request of the authors' e-mail, duplicate studies, studies from nonhuman samples including veterinary, environmental, and food products were excluded.

### 2.4. Data Screening, Extraction, and Management

To enhance screening, online records from various databases and directory were exported appropriately to ENDNOTE reference software version 8.2 (Thomson Reuters, Stamford, CT, USA). Then, the records were merged into one folder to identify and remove duplicate articles with the help of ENDNOTE or manual tracing way as there are several possibilities of citation styles per article. Then, data screening was performed by three couples of reviewers (Teklehaimanot Kiros with Lemma Workineh, Tegenaw Tiruneh with Tahir Eyayu, and Shewaneh Damtie with Debaka Belete) who independently screen the titles and abstracts of all relevant articles from literature search databases based on the predefined eligibility criteria. Authors designed a data extraction form adopted from the Cochrane collaboration and Preferred Reporting Items for Systematic Reviews and Meta-Analyses (PRISMA), 2009 checklist [14] (Supplementary file1: Table S1 PRISMA checklists) and finally customized into their study protocol to address all included studies. The data extraction format included principally first author, study ID, study design, study setting, publication year, study site in the country, sampling technique, sample size, population characteristics, the age group of study participants, the prevalence of ESBL-PE, diagnostic methods, specimen types, and types of ESBL isolates. In cases of insufficient/incomplete data, the authors independently reviewed the full-text of the article for further information and clarification. Disagreements were resolved through discussion until a consensus is reached. Then, extracted data from each article were summarized into a spreadsheet. References and data for each study were carefully crosschecked to ensure that no overlapping data were present. The study selection process was presented in a Preferred Reporting Item for Systematic Reviews and Meta-Analyses (PRISMA) flowchart [15]. Finally, a total of 18 eligible articles were included in the study.

### 2.5. Study Population

The study participants who included were all age groups, gender (male/female), and any ethnic groups living in Ethiopia.

### 2.6. Outcome Measurements

The main outcome of interest was the prevalence of ESBL-PE isolates detected from the various clinical specimens in Ethiopia.

### 2.7. Quality Assessment

Critical appraisal of the studies was made by assigned reviewers to ensure the accuracy and consistency of data. The quality of studies was assessed using standard critical appraisal tools prepared by Joanna Briggs Institute (JBI) at the University of Adelaide, Australia [16]. The main objective of the appraisal was to carefully assess the methodological quality of studies, the possibility of bias in its design and statistical analysis. The JBI appraisal checklist for prevalence studies has nine important questions. The questions (Q1–Q9) primarily focus on the appropriateness of the sampling frame, sampling techniques, the sample size, study subjects, and statistical analysis. In all cases, scores of the two authors (TK and TT) in consultation with a third author (TE) in case of discrepancy were taken for a final decision. Total scores ranged between 0 and 9. Finally, studies with a score of five and above for “yes” were included in the systematic review and meta-analysis.

### 2.8. Data Synthesis, Analysis, and Reporting

The extracted data were imported from Microsoft Excel to STATA software for the pooled estimation of outcome measures. Data manipulation and statistical analyses were performed using STATA software version 16 (College Station, Texas, USA) [17]. Der-Simonian-Laird's random-effects model was applied to estimate the pooled prevalence of ESBL-PE among the Ethiopian population at a 95% confidence level. The model is recommended to adjust for variability in the presence of heterogeneity among studies [18]. Heterogeneity was checked using *I*^2^ test statistics. *I*^2^ test statistics is the preferable and more reliable test to measure the variability across the studies. *I*^2^ ≤ 25% suggested more homogeneity, 25% < *I*^2^ ≤ 75% suggested moderate heterogeneity, and *I*^2^ > 75% suggested high heterogeneity [19]. The subgroup analysis was carried out based on the study region of studies. Finally, all statistical tests with *p* values less than 0.05 and corresponding 95% CI were considered significant. The results of the findings were presented by texts, summary tables, and figures (forest plots). This systematic review and meta-analysis were registered under PROSPERO as “CRD42019148720.”

## 3. Results

### 3.1. Characteristics of Included Studies Describing ESBL-PE

**Figure 1:**
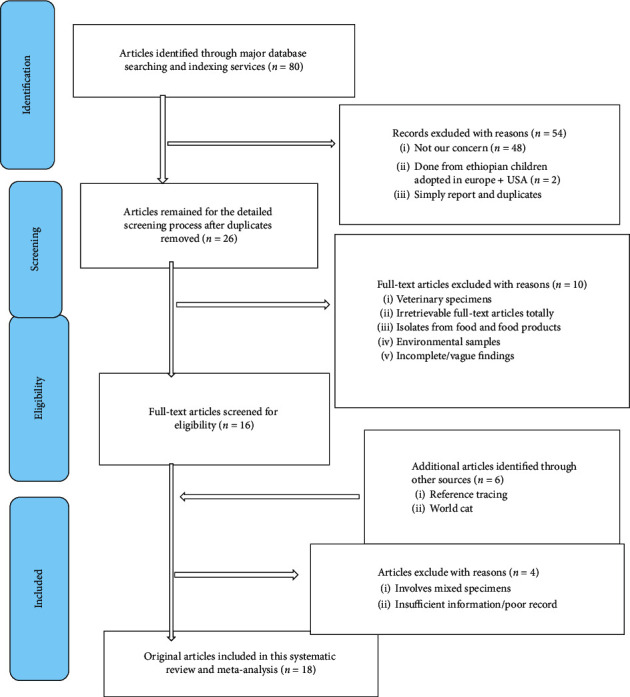
Flow diagram of studies screened and included in meta-analysis.

A comprehensive literature search was made in major electronic databases engines including Google scholar, PubMed, MEDLINE, and Web of sciences and yielded a total of 86 publications. Among the total, 68 of them were excluded after intensive reviewing of their titles, study design, outcomes, and other relevant characteristics using standard checklists and quality assessment tools (Table 1). Finally, only 18 studies (Figure 1) were potentially eligible and included in the meta-analysis. The majority of the included articles were reported from the Central Ethiopia Region (Addis Ababa, 44.4%), followed by the Oromia Region (33.3%). Not surprisingly, no reports were sought from other regions in Ethiopia such as Afar, Benishangul-Gumuz, Gambella, and Somali. The articles' year of publication has revealed that out of the total, 6/18 (33.3%) and 3/18 (16.7%) were published in the year 2018 and 2019, respectively (Table 2). Regarding the study design, the majority of the articles were conducted at a single centre with a hospital-based cross-sectional study except three articles that were multicentre cross-sectional studies involving national, regional, and private health facilities [7, 20, 21]. Around 55.6% of the studies were conducted among university hospitals including Black Lion Specialized Hospital which is the country's largest referral hospital. In this study, the most common sampling technique, 14/18 (77.8%) was the consecutive sampling technique, while two studies [22, 23] used a simple random sampling (SRS) involving both in-patient and out-patient departments. Furthermore, various clinical specimens were used by authors including blood, urine, stool, and body fluids being blood is the most common (Table 3).

### 3.2. Quality Assessment

Quality assessment for all included studies was conducted based on the JBI critical appraisal checklist. It has nine important questions (Q1–Q9) with total scores ranging from zero to nine. Studies with average quality scores ranging between five and nine were included in the systematic review and meta-analysis (Table 1).

### 3.3. Laboratory Methods Used to Detect Isolates of ESBL-PE in Ethiopia

This meta-analysis has revealed that combination disk tests (CDT) and double-disk synergy tests (DDST) were the most common diagnostic methods used by many authors to screen ESBL-producing *Enterobacteriaceae* species isolated from various human samples (Table 3). Nine out of the total (50%) reviewed articles used CDT alone to detect isolates of ESBL-PE. Likewise, 3/18 (16.7%) articles used both CDT and DDST tools. However, only 1 (5.6%) study was performed using PCR and E-test methods to detect ESBL-producing isolates. Unfortunately, none of the studies had used the broth minimum inhibitory concentration (MIC) to identify ESBL-producing Gram-negative isolates of *Enterobacteriaceae*. Regardless of the diagnostic methods utilized, only a blaCTX-M encoding gene variant was identified [22] in this review process.

### 3.4. The Pooled Estimate of ESBL-PE in Ethiopia

**Figure 2:**
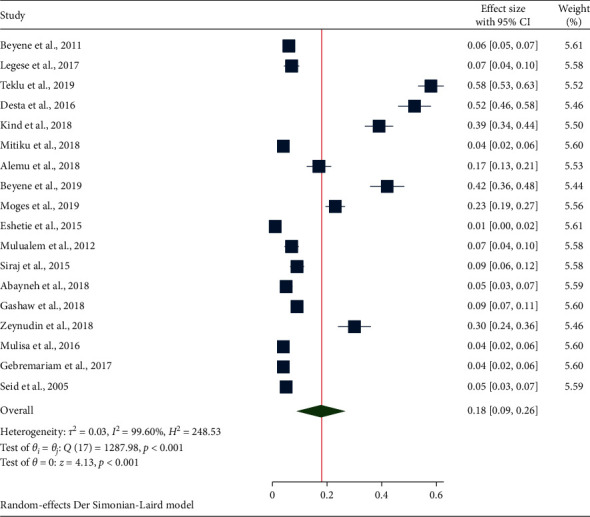
Forest plot depicting the overall prevalence of ESBL-PE in Ethiopia (2005–2019).

Based on the available included eligible studies (Figure 2), the overall pooled prevalence of ESBL-PE was found 18% (95%CI: 9–26) with a high level of heterogeneity (*I*^2^ = 99.6%, *p* < 0.001). Concerning the *β*-lactamase genes encoding to ESBL-PE, the majority, 17 (94.4%) of the studies had not determined the ESBL-encoding variant genes. Only the CTX-M gene variant was found in the single study of the included articles that determined ESBL-encoding genes.

### 3.5. Subgroup Analysis

**Figure 3:**
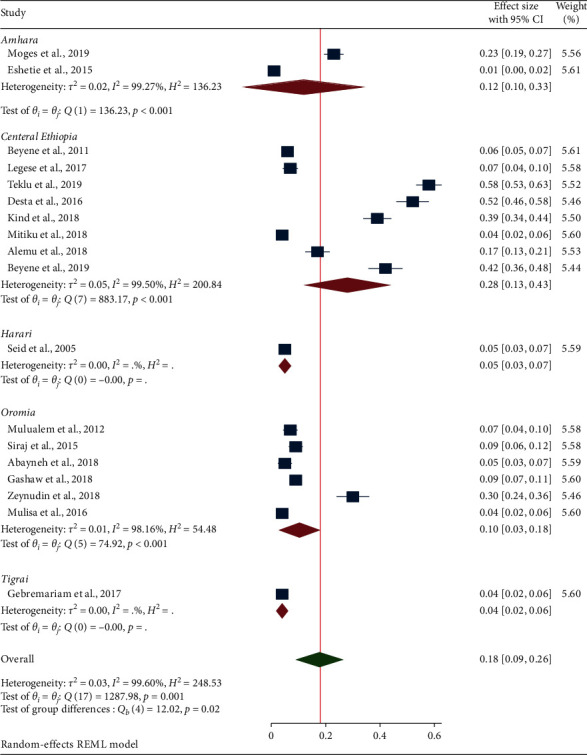
Forest plot showing the subgroup analysis of ESBL-PE based on study regions.

In this study, subgroup analysis based on the study regions was performed. Based on this, the central Ethiopia (28%) (95% CI: 13, 43) ranked the first followed by Amhara Region (12%) (95% CI: 10, 33) and Oromia Region (10%) (95% CI: 3, 18) as shown in Figure 3.

## 4. Discussion

Antimicrobial resistance especially caused by ESBL-PE has remained a major global health challenge in its many dimensions of consequences. The consequences of infection due to ESBL-PE are well known amongst many developing countries [22]. Clinical complications such as bloodstream, UTI, wound, and respiratory infections due to ESBL-PE among many developing countries are well studied. However, lack of advanced diagnostic facilities has led to increased length of hospital stay, hospital costs, poor prognosis, and even deaths [3, 8, 10, 34]. Several research findings are indicating the raise in the evolution of ESBL-PE in Africa [4, 35–39]. Studies concerning ESBL-PE among human began in 2005 in Ethiopia [21]. Despite study trends increases, none of them provides a comprehensive picture of the epidemiology of ESBL-PE conducted in the different regions of the country [6, 7, 12, 21–23, 26, 27, 29, 33, 40–43].

In the present study, a meta-analysis compiled from different scattered and limited studies within Ethiopia has revealed a pooled prevalence of 18% (95% CI: 9–26). The result is quite smaller than the nationwide survey conducted in China [44], East Africa hospitals [38], and Pakistan [45] that reported 46%, 42% (95% CI: 34–50), and 40% (95% CI: 34–47), respectively. Also, the present pooled estimate has shown relatively much smaller than other different studies conducted in the Africa region including Ghana with 49% [46], Gabon with 45% [47], Morocco with 48.4% [48], and the Asian region such as from India [49]. Moreover, substantially higher ESBL prevalence than the present study was reported from Cameroon 54% [50], Afghanistan 51.9% [51], South Korea 69.5% [52], Mali 63.4–96% [53–55], and Cameroon 82.8% [56], respectively. The current result has shown a relative consistency with previously conducted research in Germany and a report from the USA in 2012 in nine censuses that had shown in the range of 10–15% [57] and 4–12% [58] ESBL occurrence, respectively. Among the Asian continent such as in the Japanese community, an increase in ESBL-mediated resistance among *Enterobacteriaceae* was reported similar to our current ESBL prevalence where it falls between 6.3% and 20% in 9 years study [59]. Moreover, relatively concordant with the present finding was also reported by Flokas et al. [60] that was 14% (95% CI: 8–21) and a study in Africa that reported 17% (95% CI: 10–23) [35]. However, the present pooled prevalence of ESBLs among the *Enterobacteriaceae* is relatively higher than the reports including the studies by Toy et al. [39] in sub-Saharan Africa (9.3%), Flokas et al. [61] in Ghana 9% (95% CI: 6–13), Tansarli et al. [4] with <15%, and Sallem et al. [62] performed in rural areas of Africa from 2007–2012 (9.7%). The discrepancy of the present study with other studies may be explained by the differences in antibiotic-prescribing practices, the number of included studies, geographical location, and year of publication. For instance, Flokas et al. [60] had reported that the pooled ESBL-PE rate for Africa, South-East Asia, Europe, Western Pacific, Eastern Mediterranean, and in the Americas was 76% (95% CI: 56–90), 37% (95% CI: 31–43), 12% (95% CI: 2–31), 7% (95% CI: 5–9), 5% (95% CI: 1–12), and 2% (95% CI: 0–7), respectively. Also, the disparity in ESBL occurrence could be rationalized due to the variation in the socioeconomic status of a society, differences in the diagnostic performance of the applied diagnostic methods [63]. Similarly, the variation may be due to the study focus and outcome measurement [64]. For example, some studies' settings focus on community-acquired infections, whereas the other studies may concern nosocomial infections [5, 21, 37, 38, 65].

Regarding the trends in articles publication, the study prevalence of ESBL-PE over the past 15 years has grown from 5.6% to 33.33% in Ethiopia with ESBL-producing *E. coli* and *K. pneumoniae* predominant isolates. In a similar fashion in Tunisia in the study periods from 1999 to 2012, the prevalence of ESBL-PE increased from 11.7% to 77.8% [62, 66–69]. Similarly, a Chinese study in 2012 had reported that ESBL-producing *E. coli* isolate was 52.2% [70], while it was 1.6% in Hong Kong in 1990 and 2.6% in 1995. Meanwhile, the proportion in *Klebsiella* spp. and *Enterobacter* spp. were estimated at 3% and 10% and 24% and 23% in 1990 and 1995, respectively [71]. Moreover, a study in India has shown that ESBL-producing *E. coli* increased from 40% to 61% in the study period of 2002–2009. But, the prevalence of ESBL-producing *K. pneumoniae* remained almost stable, ranging from 38%–40% in the study period of 2002–2009 [72].

In this study, the most commonly used clinical specimen used by the majority of the authors was blood followed by urine, body discharges, and stool. This is similar to the studies from South Korea [52], Tanzania [73], Algeria [4, 35, 39, 74–76], Nigeria [77], Mali [53, 54], Niger [78], and Cameroon [56]. In this review, more than 90% of the studies were conducted on health facilities, and three of them were performed among multicentre health intuitions [7, 20, 21]. Several studies have also shown that the community prevalence of ESBL-PE is lower than the hospital-based prevalence such as in studies in Tunisia that ranges 0.7–7.3% in community and 11.7–77.8% in hospital settings, and in Egypt, ranges 11–42.9% from the community as well as from hospital settings [62, 79]. Similarly, in Kenya [34], Ghana [80], and South African [36] were reported 37.4%, 49.4%, and 0.3–13% prevalence from the community and hospital settings, respectively. On the other hand, slightly higher community proportion estimates (18.8%) than hospital settings (16.4%) have been reported from South America [81, 82]. The disparity in ESBLs occurrence can be influenced by the choice in the diagnostic method.

Various ESBL identification methods were used in these reviewed articles such as the double-disk synergy test, combination disk test, polymerase chain reaction (PCR), and E-test. But other similar studies abroad used pulsed-field gel electrophoresis and other molecular techniques [10, 39, 60, 83]. In the majority of reviewed articles, nonmolecular tests including the combination disk test, double-disk synergy test, and E-test were the most widely used tests across the different regions of the country to screen ESBL isolates from heterogeneous clinical specimens. That means a lack of advanced diagnostic tools such as PCR and DNA sequencing in Ethiopia has remained the major problem to address the common variant ESBL-encoding genes. Not only in Ethiopia but also among several Africa countries, the subdetection tests are primarily used for the identification of responsible strains during ESBL epidemics both in a hospital as wells as in a community setting [39, 45, 84]. Hence, the difference in the diagnostic test is considered as a contributing factor to the clinical disparity of ESBL proportion [10, 21, 45, 85]. For this reason, only one study [31] had used PCR sequencing, while the rest were performed using purely phenotypic screening tools. In the present study, the only ESBL gene was CTX-M. However, many documents have shown that class “A” ESBL gene is the most frequently encountered genes in hospital and community settings [10, 41]. For instance, the CTX-M-15 gene is identified in many studies and is usually associated with other types of genes such as CTX-M [64], TEM, and SHV [64]. The CTX-M group was sought and considered prevalent in 50% of studies that had performed ESBL strain characterization using PCR-based molecular detection methods [82, 86].

### 4.1. Strengths and Limitations of This Study

This study provides a general picture of the prevalence of ESBL-PE in Ethiopia. Since it is the first meta-analysis study in the country, it is expected to provide local knowledge for the healthcare providers and health policy makers in general. However, due to the lack of published studies from some regions or locations of Ethiopia, the estimate of the outcome measures may not represent a national figure for the burden of AMR due to ESBL-PE. Furthermore, this study primarily focuses on ESBL-producing organisms detected from human specimens. But, isolates from nonhuman origins are not addressed which were also considered another limitation for this study.

## 5. Conclusion

In conclusion, the pooled prevalence of ESBL-PE detected from the various clinical specimens is relatively low in Ethiopia. This meta-analysis has shown an echo for ESBL isolates in Ethiopia. AMR especially caused by ESBL-producing isolates of *Enterobacteriaceae* has remained a global public health problem. The research in gab particularly with the gene variants encoding to ESBL-PE calls for integrated active surveillance systems which can support to summarize and elucidate the ongoing epidemiology picture of ESBLs in Ethiopia. Furthermore, strategic interventions to combat antimicrobial resistance including effective infection prevention and control and rational use of antibiotics should be implemented by using the data even from other parts of the globe to contain further spread of the ESBL-PE. Decisive measures have to be taken to stop the higher colonization rate with nosocomial ESBL-PE; otherwise, the use of carbapenems to treat community and nosocomial infections will subsequently result in the emergence of carbapenemase-producing pathogens.

## Figures and Tables

**Table 1:** Quality assessment of studies using JBI's critical appraisal tools designed for cross-sectional studies, Ethiopia (2005–2019).

Authors and reference	9-Point Joanna Briggs Institute (JBI) critical appraisal tools									Overall score	Included
	Q1	Q2	Q3	Q4	Q5	Q6	Q7	Q8	Q9		
Beyene et al. [20]	Y	Y	Y	Y	Y	Y	Y	Y	Y	9	✓
Alemu [24]	Y	Y	Y	Y	Y	Y	Y	N	Y	8	✓
Legese et al. [6]	Y	Y	Y	Y	Y	Y	Y	Y	Y	9	✓
Teklu et al. [7]	Y	Y	Y	Y	Y	Y	Y	Y	Y	9	✓
Desta et al. [12]	Y	Y	Y	Y	Y	Y	Y	U	Y	8	✓
Beyene et al. [25]	Y	Y	Y	N	Y	Y	N	Y	Y	7	✓
Kind [26]	N	Y	Y	Y	U	Y	Y	N	Y	6	✓
Mitiku [27]	N	Y	Y	Y	N	Y	Y	Y	N	6	✓
Moges et al. [28]	Y	Y	Y	Y	Y	Y	Y	Y	Y	9	✓
Eshetie et al. [23]	Y	Y	Y	Y	Y	Y	Y	Y	Y	9	✓
Mulualem et al. [29]	Y	Y	N	Y	Y	Y	Y	N	Y	7	✓
Abayneh et al. [30]	Y	Y	Y	N	Y	Y	Y	Y	Y	8	✓
Gashaw et al. [31]	Y	Y	Y	Y	Y	Y	Y	Y	Y	9	✓
Zeynudin et al. [22]	Y	Y	Y	Y	Y	Y	Y	Y	Y	9	✓
Siraj et al. [11]	Y	Y	Y	U	Y	Y	N	Y	Y	7	✓
Mulisa et al. [32]	Y	N	Y	Y	Y	Y	Y	Y	Y	8	✓
Gebremariam et al. [33]	U	Y	Y	N	Y	Y	Y	Y	Y	7	✓
Seid and Asrat [21]	Y	Y	Y	Y	Y	N	Y	Y	Y	8	✓

Y, yes; N, no; U, unclear; Q, question. The overall score is calculated by counting the number of Y's in each row (score of five and above were included in the systematic review and meta-analysis). Q1, Was the sample frame appropriate to address the target population? Q2, Were study participants sampled in an appropriate way? Q3, Was the sample size adequate? Q4, Were the study subjects and the setting described in detail? Q5, Was the data analysis conducted with sufficient coverage of the identified sample? Q6, Were valid methods used for the identification of the condition? Q7, Was the condition measured in a standard reliable way for all participants? Q8, Was there appropriate statistical analysis? Q9, Was the response rate adequate, and if not, was the low response rate managed appropriately?

**Table 2:** Distribution and characteristics of studies on ESBL-PE in Ethiopia (2005–2019).

Authors and reference	Study area	Region	Study design	Population
Beyene et al. [20]	Addis Ababa	Central Ethiopia	MC-cross-sectional	Children with febrile illness and diarrheal diseases
Alemu [24]	Addis Ababa	Central Ethiopia	Cross-sectional	<5 Children suspected for colonization/carriage
Legese et al. [6]	Addis Ababa	Central Ethiopia	Cross-sectional	Children suspected of septicemia and UTIs
Teklu et al. [7]	Addis Ababa	Central Ethiopia	MC-cross-sectional	Patients
Desta et al. [12]	Addis Ababa	Central Ethiopia	Cross-sectional	Hospitalized patients with gastrointestinal colonization
Beyene et al. [25]	Addis Ababa	Central Ethiopia	Cross-sectional	Patients
Kind [26]	Addis Ababa	Central Ethiopia	Cross-sectional	Patients
Mitiku [27]	Addis Ababa	Central Ethiopia	Cross-sectional	Septicemia suspected
Moges et al. [28]	Bahir Dar	Amhara	Cross-sectional	All patients suspected of UTI and other infections
Eshetie et al. [23]	Gondar	Amhara	Cross-sectional	UTI suspected patients
Mulualem et al. [29]	Jimma	Oromia	Cross-sectional	Patients suspected of UTI and GIT
Abayneh et al. [30]	Jimma	Oromia	Cross-sectional	Patients suspected of community-onset UTI
Gashaw et al. [31]	Jimma	Oromia	Cross-sectional	Patients suspected of HAIs
Zeynudin et al. [22]	Jimma	Oromia	Cross-sectional	Patients suspected of UTI wound infections
Siraj et al. [11]	Jimma	Oromia	MC-cross-sectional	Patients suspected of UTI, wound infections, GIT, and respiratory infections
Mulisa et al. [32]	Adama	Oromia	Cross-sectional	Patients
Gebremariam et al. [33]	Mekelle	Tigray	Cross-sectional	University students
Seid and Asrat [21]	Harrar	Harrari	MC-cross-sectional	Admitted patients

MC, multicentre; UTI, urinary tract infection; GIT, gastrointestinal tract infection; HAIs, hospital-acquired infections In the total of 18 included studies, a total of 1191 bacterial isolates were recovered from 7919 various clinical samples being *E. coli* and *K. pneumoniae* were the most studied isolates of ESBL-PE accounting for 50% followed by the combination of other species such as *Proteus* spp., *K. oxytoca, E. cloacae, Citrobacter* spp., *E. aerogenes, Salmonella* spp., and *C. freundii* with *E. coli* and *K. pneumoniae* yielding 38.9% prevalence.

**Table 3:** Clinical characteristics of included articles describing ESBL-PE in Ethiopia (2005–2019).

Studies	Sample size	Clinical specimen	Diagnostic method	Bacterial species	No. of ESBL (%)
Beyene et al. [20]	1225	Stool and blood	E-test	*S. concord*	78 (6.4)
Legese et al. [6]	322	Urine and blood	CDT and DDST	*K. pneumoniae* and *E. coli*	22 (7)
Teklu et al. [7]	426	Pus, urine, blood, CSF, and sputum	CDT and DDST	*K. pneumoniae* and *E. coli*	246 (58)
Desta et al. [12]	267	Stool	CDT	*K. pneumoniae* and *E. coli*	139 (52.1)
Kind [26]	338	Stool, urine, sputum, body fluid, and pus	CDT	*K. pneumoniae, E. coli,* and others^#^	131 (39)
Moges et al. [28]	532	Blood, urine, stool, body fluid, eye discharges, and wound swab	CDT	*K. pneumoniae* and others^#^	121 (23)
Eshetie et al. [23]	442	Urine	DDST	*K. pneumoniae* and *E. coli*	5 (1.13)
Mulualem et al. [29]	359	Urine, sputum, stool, and wound swab	DDST	*E. coli*	24 (7)
Abayneh et al. [30]	342	Urine	CDT	*K. pneumoniae* and *E. coli*	17 (5)
Gashaw et al. [31]	1015	Blood, urine, pus, sputum, and wound swab	E-test and PCR	*K. pneumoniae, E. coli,* and others^#^	89 (9)
Zeynudin et al. [22]	224	Urine and wound swab	CDT	*E. coli* and others^#^	68 (30.4)
Siraj et al. [11]	471	Urine, sputum, pus, blood, eye discharge, and vaginal swab	CDT	*K. pneumoniae* and *E. coli*	43 (9)
Mulisa et al. [32]	384	Urine, stool, and body fluid	CDT	*K. pneumoniae* and others^#^	17 (4.4)
Gebremariam et al. [33]	341	Urine	DDST	*K. pneumoniae* and *E. coli*	12 (3.5)
Seid and Asrat [21]	384	Sputum, urine, and pus	DDST	*K. pneumoniae*	19 (5)
Mitiku [27]	340	Blood	CDT and DDST	*K. pneumoniae* and *E. coli*	15 (4.4)
Alemu [24]	269	Stool and rectal swab	CDT	*K. pneumoniae* and *E. coli*	46 (17.1)
Beyene et al. [25]	238	Urine, sputum, pus, blood, eye discharge, and body fluid	CDT	*K. pneumoniae*, *E. coli*, and others^#^	99 (42)

E-test, epsilometric test; CDT, combination disk test; DDST, double-disc synergy test; PCR, polymerase chain reaction. ^#^Others include *Proteus* spp., *K*. *oxytoca, E. cloacae, Citrobacter* spp, *E. aerogenes, Salmonella* spp., *Shigella* spp., *Serratia* spp., *Providencia stuartii,* and *Morganella* spp.

## Data Availability

The data used to support the findings of this study are included within the article.

## Abbreviations

AMR: Antimicrobial resistance
CDT: Combination disk test
DDST: Double-disc synergy test
ESBL-PE: Extended-spectrum beta-lactamase-producing *Enterobacteriaceae*
PCR: Polymerase chain reaction
UTI: Urinary tract infections.

## Supplementary Materials

Supplementary Materials Table S1: supporting information for PRISMA checklists.
